# Clinical profile, evaluation, management and visual outcome of idiopathic intracranial hypertension in a neuro-ophthalmology clinic of a tertiary referral ophthalmic center in India

**DOI:** 10.4103/0972-2327.61275

**Published:** 2010

**Authors:** S. Ambika, Deepak Arjundas, Veena Noronha, Anshuman

**Affiliations:** Department of Neuro Ophthalmology, Medical Research Foundation, Sankara Nethralaya, Chennai, India

**Keywords:** Cerebrospinal fluid opening pressure, lumboperitoneal shunt, modified Dandy's criteria, optic disc edema

## Abstract

**Aim:**

To discuss the clinical features and management of patients who presented with optic disc edema and had features of presumed idiopathic intracranial hypertension (IIH).

**Materials and Methods:**

Case series of all patients diagnosed to have IIH from January 2000 to December 2003 in the neuro-ophthalmology clinic of a tertiary referral ophthalmic institution, were retrospectively analyzed. Analysis was done for 50/106 patients who fulfilled modified Dandy's criteria and had optic disc edema and a minimal follow-up period of two years.

**Results:**

Most (40/50, 80%) of the patients were females and the mean age of presentation for all the 50 patients was 32.89 years. Chief complaints were headache in 38 (76%) patients, 24(48%) patients had transient visual obscuration, 24 (48%) patients had reduced vision, 15 (30%) patients had nausea, vomiting, 4(8%) patients had diplopia. Bilateral disc edema was seen in 46 (92%) patients and unilateral disc edema in 4 (8%) patients. 60 eyes had enlarged blind spot as the common visual field defect. Neuroimaging revealed prominent perioptic CSF spaces in 14 patients and empty sella in three patients. CSF opening pressure was 250–350 mm H2O (water) in 39 patients and was >350 mm H2O in 11 patients. Medical treatment was started for all patients; whereas 35 [70%] patients responded, 15 [30%] patients had to undergo LP shunt.

## Introduction

Benign [idiopathic] intracranial hypertension is a disease defined by elevated cerebrospinal fluid pressure, normal cerebrospinal fluid content, a normal brain with normal or small ventricles seen in imaging studies, and normal results of neurologic examination except for abducens nerve palsy (modified Dandy's criteria).[1–3]

This condition has been well described in adults, with a strong female preponderance and an association with obesity. Although these cases are mostly diagnosed and managed by neurologists, they also present to an ophthalmologist at times without any alarming neurological symptoms.

## Materials and Methods

A retrospective chart analysis was done for patients with papilledema who presented in the neuro-ophthalmology services of our institution, a tertiary referral center for ophthalmology, between January 2000 and December 2003.

Inclusion criteria:

1. Unilateral or bilateral disc edema of any grade,
2. A nonfocal neurological examination except for isolated abducens nerve palsy
3. Normal brain magnetic resonance imaging and/or computed tomographic scan
4. CSF pressure >200 mm of water with normal cerebrospinal fluid content [1]

Patients with concurrent ocular illness were excluded

Modified Dandy's Criteria for the diagnosis of idiopathic intracranial hypertension:

1. Signs and symptoms of increased intracranial pressure
2. Absence of localizing findings on neurological examination
3. Absence of deformity, obstruction, and displacement of the ventricular system and otherwise normal neurodiagnostic studies except for increased cerebrospinal fluid pressure (> 200 mm of water in nonobese patients and >250 mm of water in obese patients)
4. Awake and alert patient
5. No other cause for increased intracranial pressure

A detailed history was taken, including demographic features of age and gender, date of presentation, presenting symptoms of headache, nausea, vomiting, neurological symptoms, and ocular symptoms like transient visual loss, loss of central vision, visual field defects, and double vision. [4] The presence and duration of any systemic disease (e.g. hypertension), relevant drug history (e.g., systemic corticosteriods, oral contraceptive pills, and vitamins). A detailed ophthalmic examination was performed, including best corrected snellen visual acuity, refraction, ocular motility examination, pupil evaluation, color vision, anterior segment examination, intraocular pressure measurement, optic disc examination with +90 D biomicroscopy, and fundus examination with binocular indirect ophthalmoscopy and +20 D lens. Humphrey's 30-2 visual field analysis, neuroimaging (either a CT scan or a MRI scan with MR venography in some patients), and lumbar puncture: cerebrospinal fluid (CSF) analysis and pressure recording was done for all patients. Upon diagnosis of all features of IIH, patients were initially assigned to Group 1: medical treatment consisting of carbonic anhydrase inhibitors (e.g., acetazolamide) that decrease CSF secretion by the choroid plexus. [5] Acetazolamide [1–2 g/day] was used for all patients but its dosage was reduced in patients who were not able to tolerate the side effects (i.e., nausea, vomiting, sedation, and metabolic acidosis). Along with this, all patients were treated with intravenous methyl prednisolone 1 g/day ×5 days[6] followed by oral steroids over a period of four weeks. As long-term steroid therapy can cause raised intracranial pressure, its usage was limited for only a brief period. Patients were assigned to Group 2: surgical treatment (CSF diversion procedures) if the Group 1 medical treatment did not show any improvement in Snellen's visual acuity [minimum two lines] or visual field defects with persisting disc edema for 6–8 weeks. Visual acuity, optic disc changes, and visual field defects were checked in all patients during follow-up. Patients were followed up weekly for a month, monthly for three months and after every six months for two years.

## Results

Out of 106 patients with IIH, 50 patients fulfilled the inclusion criteria stated above. Out of those 50 patients, 40 were females (female: male ratio 4:1) and the average age of onset was 32.89 years (range: 3½ to 49 yrs); all patients were symptomatic.

The most common presenting symptom was headache (38 patients, 76%)[4], followed by reduced visual acuity (24 patients, 48%), transient visual obscurations (24 patients, 48%), nausea and vomiting (15 patients, 30%), and diplopia (four patients, 8%). Nineteen patients were hypermetropic, 16 were myopic, and 15 emmetropic.

**Figure 1:**
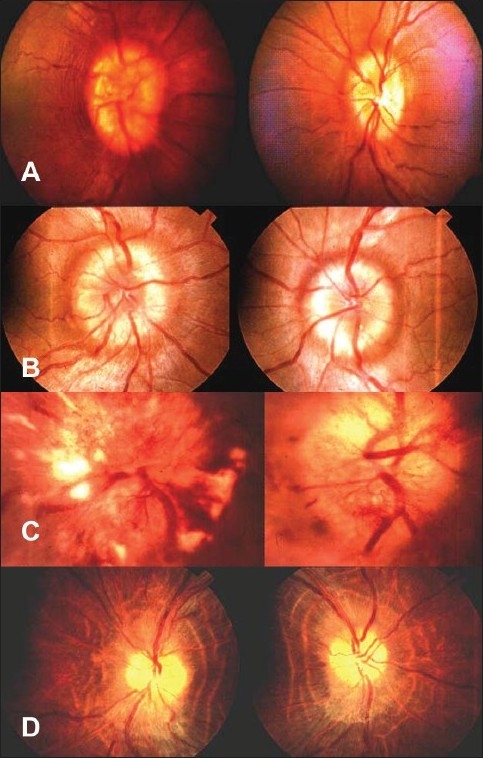
Fundus pictures showing (A) early papilledema (B) established papilledema (C) vintage papilledema and (D) atrophic papilledema

On presentation, best corrected visual acuity was found to range from 6/6 to perception of light.[7] Fifty-five eyes had 6/6 visual acuity, 20 eyes had 6/9–6/18 visual acuity, seven eyes had 6/24–6/60, and 18 eyes had acuity <6/60. Color vision analysis using an Ishihara chart showed that color vision was normal [16/16] in 14 patients but was affected in ten patients; it was not done in 26 patients. Bilateral disc edema was noted in 46 patients (92%) whereas unilateral disc edema was seen in four patients (8%). Patients also presented with features of early to established papilledema [Figure 1A and B]. Most patients (70%) were found to respond to treatment, which decreased disc edema whereas the remaining 30% patients had gone into stages [Figure 1C and D] of vintage and atrophic papilledema stages without any improvement.

**Figure 2:**
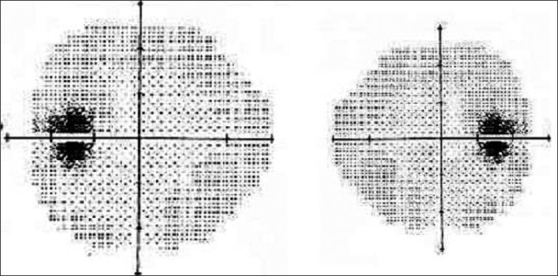
Perimetry showing bilateral enlarged blind spot

The most common visual field defect was an enlarged blind spot [Figure 2], which was noted in 60 eyes Other field defects were advanced generalized constricted fields (11 eyes), as well as nasal and arcuate defects (seven eyes). Twelve eyes had normal visual fields but fields could not be done in ten eyes (the patients were too young or had poor vision).

**Figure 3:**
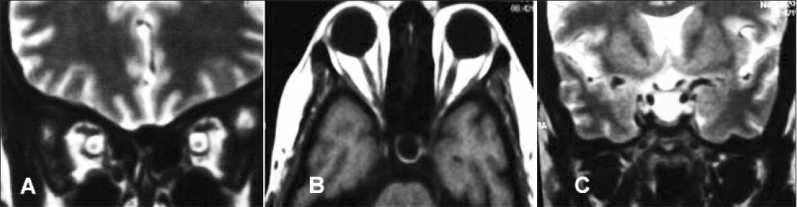
MRI images coronal section through brain and orbit showing widening of the perioptic space (A) and empty sella in axial section T1 sequence (B) coronal section T2 sequence (C)

MRI of the brain and orbit was done in 42 patients out of whom 25 patients were found to be normal whereas the remaining 17 patients were found to have BIH features. Perioptic space widening (14 patients) and empty sella (three patients) [Figure 3A–C] are pathognomonic MRI features that are suggestive of BIH. MR venogram was done in 19 patients and all were found to be normal. None of our patients had cerebral venous thrombosis.[8] CT of the brain alone was done in eight patients. MRI + MRV is the study of choice for IIH patients.

Lumbar puncture and CSF analysis (CSF opening and closing pressure recordings) were done for all patients. CSF composition was observed to be normal in all patients.[9] Thirty- nine patients had opening pressure of 250–350 mm H2O and 11 patients had opening pressure >350 mmH2O

### Treatment

Results

All 50 patients were medically treated out of which 35 [70%] patients showed signs of improvement—visual acuity improved by two lines in Snellen's chart, disc edema had decreased, field defects had improved, and they had maintained their 6/6 vision [Table 1]. They were all in early to established stages of papilledema. The remaining 15 [30%] patients did not show any clinical improvement even after four weeks and hence, were advised to go for CSF diversion procedures [lumboperitoneal / ventriculoperitoneal shunts]. They showed only moderate recovery in their visual fields and 1–2 lines of vision improvement in Snellen's visual acuity chart even after eight weeks in their subsequent follow-up.[10]

**Table 1:** Summary of clinical outcome of persons with idiopathic intracranial hypertension

No. of eyes	On presentation Visual acuity	ONH appearance	Visual fields	Treatment	Follow-up 3 mo	Follow-up 6 mo
55 eyes	6/6	Early disc edema	EBS Normal	Medical	6/6, HVF and disc N	6/6, disc and HVF –N
20 eyes	6/9–6/18	Early–established disc edema	EBS Arcuate	Medical	6/9–6/12 EBS, mild Disc edema	6/6, disc min pallor, fields GRS
7 eyes	6/24–6/60	Established disc edema	GRS Arcuate	Medical	6/24–6/36 GRS, disc edema decreasing	6/18-6/24 Pale disc GRS
18 eyes	< 6/60	Vintage edema, Atrophic	Constricted fields	Medical+ surgical	<6/60-VA Pale disc Constricted Fields	<6/6/0 VA Optic atrophy Constricted fields

EBS = Enlarged blind spot, GRS = Generalied reduction of sensitivity, HVF = Humphrey visual field, VA = Visual acuity

Of these 15 patients who were surgically treated, seven patients underwent shunt surgery of whom three patients showed signs of improvement by 3–4 weeks. Vision deteriorated in two patients but remained the same in the remaining two patients even at the end of six weeks. Eight out of the 15 surgically treated patients were subsequently lost to follow-up.

## Discussion

Although the syndrome of increased intracranial pressure without ventriculomegaly or mass lesion, but normal cerebrospinal fluid (CSF) composition, was first described over a century ago, we still know very little about its pathogenesis. Often referred to as, “pseudotumor cerebri,” it is more appropriately called, “idiopathic intracranial hypertension.”[1]

Pathogenesis of pseudotumor cerebri is unclear,[11] but decreased CSF absorption by the arachnoid villi is the most commonly suggested explanation. Elevated intracranial venous pressure causes delayed absorption of the CSF. Obesity causes raised intraabdominal pressure that results in increased pleural and cardiac filling pressure, leading to raised intracranial venous pressure and ultimately, increased CSF pressure.[5,12]

The typical IIH patient is an obese woman of childbearing age. Atypical patients include men, slim women, prepubertal children, and patients older than 44 years. In a prospective study of 50 patients, 92% were women with a mean age of 32 years, with obesity seen in 92% of the patients.

The most common symptoms of IIH include headache, transient obscuration of vision, pulsatile tinnitus, and diplopia. In a prospective study[9] of 50 idiopathic IIH patients, symptoms included headache (94%), transient visual obscuration (68%), intracranial noises (58%), sustained visual loss (26%), photopsia (54%), diplopia (38%), and retrobulbar pain (44%).

Often, the IIH patient is unaware of peripheral visual field dysfunction and Snellen's acuity testing is a poor indicator of early visual deficit. Rowe and Sarkies[7] studied 35 IIH patients prospectively and found that visual field assessment was the most sensitive indicator of visual loss with a statistically greater sensitivity than visual acuity or contrast sensitivity testing.

The most common field defects are enlarged blind spots, arcuate defects, nasal steps, and global constriction of the visual fields. Although unilateral / asymmetrical papilledema is frequently found in IIH, most of our patients suffered from bilateral and symmetric disc swelling.

A noncontrast CT was earlier considered as an adequate imaging study because it could exclude ventriculomegaly or mass lesion. However, conditions that increase ICP without producing ventriculomegaly or mass lesions, such as gliomatosis cerebri, meningitis, and cerebral venous thrombosis, may mimic IIH and yet not have associated CT abnormalities to provide a clue to the true underlying condition. Unless there are external constraints (weight limitations, availability), MRI with MRV is currently the study of choice.[13]

Using a special technique, three-dimensional, gadolinium- enhanced MRV appears to be more sensitive than conventional MRV for detecting areas of subtle cerebral venous stenosis.

Medical literature contains various, and sometimes conflicting, recommendations regarding the minimum CSF opening pressure required for diagnosing IIH. In general, however, a CSF pressure >250 mm of water is consistent with a diagnosis of IIH, <200 mm of water is considered normal, and 201–249 mm of water is considered to be nondiagnostic. Contrary to popular belief, there is no evidence that body weight influences these cutoff values.[5] The upper limit of CSF pressure in children is generally considered to be 180–200 mm of water and the effect of obesity in children has not been studied.

Patients are treated in the presence of visual acuity or visual field loss (apart from mild enlargement of a blind spot), moderate to severe papilledema, or persistent headaches associated with a significant rise in intracranial pressure.

Dietary management and weight loss are time-honored treatments, supported by several observational studies. There is little scientifically robust information regarding specific dietary measures for IIH.

A few retrospective studies[5] have documented that IIH improved following diet restriction, gastric bypass surgery etc.

Repeated lumbar punctures are sometimes used in patients with occasional symptomatic relapses, in pregnant women, or in the setting of rapidly declining vision to temporarily lower the CSF pressure while planning a more aggressive treatment. However, the procedure may be painful, technically difficult to perform, and cause a low-pressure headache; oher complications are rare. Considering that 50 mL of CSF is produced in a day in humans at a rate of approximately 0.35 mL/min, 20 mL of CSF can be removed by lumbar puncture is replenished in one hour, provided there is no persistent CSF loss through the dural puncture site or alteration in CSF production caused by lumbar puncture.

Acetazolamide is generally accepted as a first-line medication for lowering the intracranial hypertension in IIH patients. Its carbonic anhydrase inhibition decreases the secretion of CSF by the choroid plexus. Doses of 1–2 g are generally used and some advocate increasing to the maximum tolerated dose if necessary. Side effects are common but are better accepted when patients are aware of their potential occurrence and medication doses are built up gradually.

In our series, medical treatment included a combination of systemic corticosteroids (intravenous methylprednisolone followed by oral steroids), a systemic carbonic anhydrase inhibitor (oral acetazolamide), systemic hyperosmotic agents for a few cases (intravenous mannitol and oral glycerol).[6]

Corticosteroids are not advocated for routine or long-term management of IIH. They are, however, useful as an adjunctive treatment in patients in whom rapid deterioration is seen while arranging a surgical procedure. Withdrawal of steroids may lead to a rebound increase in ICP and their side-effects can be problematic in IIH patients.

Indications for surgery include progressive loss of vision despite maximal medical therapy, severe or rapid visual loss at onset (including the development of afferent papillary defect or signs of advancing optic nerve dysfunction), and severe papilledema causing macular edema or exudates.

Surgical procedures include optic nerve sheath decompression (ONSD) and CSF diversion procedures. It is controversial as to which procedure is superior and the decision often depends on available resources and expertise.

A CSF diversion procedure treats IIH by lowering intracranial pressure but requires the insertion of a foreign body.[14]

Lumboperitoneal shunting[10] is more commonly performed than ventriculoperitoneal shunting because insertion and maintenance of patency may be more difficult in the latter procedure. The revision rate for lumboperitoneal shunt ranges from 38 to 64%, with an overall revision rate of 52%. The reported interval between shunt placement and first revision is 9–27 months.

In our series, seven [10%] patients underwent lumboperitoneal shunt procedure because of their lack of response to medical therapy. The maximum follow-up in these patients was 11 months, and they did not need revision until then.

## Conclusion

Benign intracranial hypertension is a common cause of papilledema in young, overweight females. CSF pressure recording is mandatory in these patients and early treatment can only help salvaging the vision. In our series, we have found that early onset [relatively good visual acuity, early disc edema, and normal/minimal field defects] of medical treatment has prevented them from ending up with irreversible vision loss and optic atrophy.
